# Gene Therapy Model of X-linked Severe Combined Immunodeficiency Using a Modified Foamy Virus Vector

**DOI:** 10.1371/journal.pone.0071594

**Published:** 2013-08-21

**Authors:** Satoshi Horino, Toru Uchiyama, Takanori So, Hiroyuki Nagashima, Shu-lan Sun, Miki Sato, Atsuko Asao, Yoichi Haji, Yoji Sasahara, Fabio Candotti, Shigeru Tsuchiya, Shigeo Kure, Kazuo Sugamura, Naoto Ishii

**Affiliations:** 1 Department of Microbiology and Immunology, Tohoku University Graduate School of Medicine, Sendai, Japan; 2 Department of Pediatrics, Tohoku University Graduate School of Medicine, Sendai, Japan; 3 Genetics and Molecular Biology Branch, National Human Genome Research Institute, National Institutes of Health, Bethesda, Maryland, United States of America; 4 Miyagi Cancer Center, Natori, Japan; University of Hawaii, United States of America

## Abstract

X-linked severe combined immunodeficiency (SCID-X1) is an inherited genetic immunodeficiency associated with mutations in the common cytokine receptor γ chain (γc) gene, and characterized by a complete defect of T and natural killer (NK) cells. Gene therapy for SCID-X1 using conventional retroviral (RV) vectors carrying the γc gene results in the successful reconstitution of T cell immunity. However, the high incidence of vector-mediated T cell leukemia, caused by vector insertion near or within cancer-related genes has been a serious problem. In this study, we established a gene therapy model of mouse SCID-X1 using a modified foamy virus (FV) vector expressing human γc. Analysis of vector integration in a human T cell line demonstrated that the FV vector integration sites were significantly less likely to be located within or near transcriptional start sites than RV vector integration sites. To evaluate the therapeutic efficacy, bone marrow cells from γc-knockout (γc-KO) mice were infected with the FV vector and transplanted into γc-KO mice. Transplantation of the FV-treated cells resulted in the successful reconstitution of functionally active T and B cells. These data suggest that FV vectors can be effective and may be safer than conventional RV vectors for gene therapy for SCID-X1.

## Introduction

X-linked severe combined immunodeficiency (SCID-X1) is a life-threatening immunodeficiency disorder, characterized by defective T and natural killer (NK) cell production and the development of functionally impaired B cells that lack the capacity to produce immunoglobulins. These defects result in a profound reduction in the development of both cellular and humoral immunity. SCID-X1 is caused by inactivating mutations in the gene encoding the cytokine receptor γ chain (γc), a common subunit of the receptors for interleukin (IL)-2, IL-4, IL-7, IL-9, IL-15, and IL-21 [1]. Bone marrow transplantation (BMT) from human leukocyte antigen (HLA)-identical siblings can cure the disease with a success rate of approximately 90%. However, BMT from non-HLA-identical donors results in lower survival rates due to a high risk for complications such as graft-versus-host disease, graft rejection, and incomplete T cell engraftment [2], [3]. Consequently, gene therapy approaches have been developed as an alternative treatment option for those patients lacking appropriate donors.

The first gene therapy clinical trial for SCID-X1 was carried out by a French group in 1999 [4], [5]. In that study, a conventional retroviral (RV) vector expressing γc was used, and resulted in the reconstitution of T and NK cell populations, and the recovery of humoral immunity. However, over time, acute T cell leukemia developed in 5 of the 20 patients receiving the therapy. The leukemia cells of these patients showed aberrant and high expressions of proto-oncogenes such as *LMO2*, which were caused by RV insertions within or near these loci [4], [5]. To reduce the risk of vector-mediated insertional mutagenesis, various types of new vectors have since been developed [6], .

Foamy virus (FV) is a non-pathogenic retrovirus belonging to the spumavirus genus and has unique biological characteristics, such as a wide host range (including humans), and wide tissue tropism [8], [9]. Refined FV vectors that have large packaging capacities and are able to transduce murine and human hematopoietic stem cells (HSCs) have been reported [10]–[12]. In addition, FV vectors are reported to have a reduced tendency to integrate within or adjacent to the coding regions of genes compared to RV vectors. Due to these advantages, FV vectors have recently been used to correct genetic deficiencies in hematopoietic stem cells (HSCs) in several mouse models; these diseases include Wiskott–Aldrich syndrome (WAS) [13], leukocyte adhesion deficiency [14], Fanconi anemia [15], β-thalassemia [16], and X-linked chronic granulomatous disease [17].

In the present study, we evaluated the rate of insertional mutagenesis by a γc-FV vector compared to that of an RV vector in human T cells, and demonstrated the effectiveness of the γc-FV vector in a murine gene therapy model of SCID-X1.

## Methods

All procedures were performed according to the protocols approved by the Institutional Committee for Use and Care of Laboratory Animals of Tohoku University, which was granted by Tohoku University Ethics Review Board (No. 2010MA165) and the Guide for Care and Use of Laboratory Animals published by the U.S. National Institutes of Health (NIH publication 85–23, revised 1996).

### FV vector construction and production

**Figure 1:**
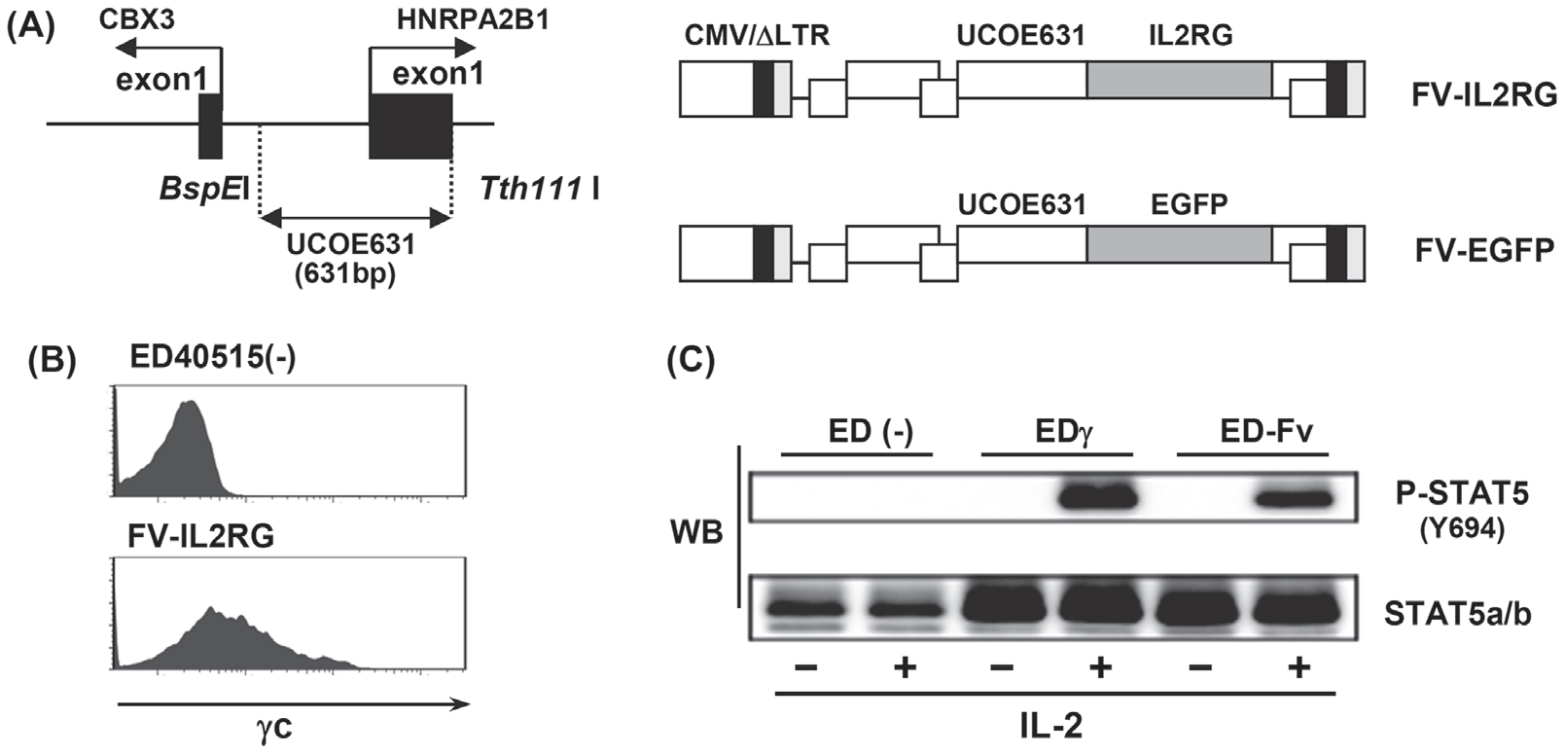
Structure and *in vitro* analysis of foamy virus vectors. (**A**) Structure of the foamy virus vectors. The UCOE631 promoter sequence from the human HNRPA2B1-CBX3 locus and transgenes were inserted into the FV vector. (**B**) Cell-surface expression of γc on ED40515(–) cells transduced with the FV-IL2RG vector. (**C**) STAT5 phosphorylation upon IL-2 stimulation. ED40515(–) cells, an ED40515(–)-derived transfectant with a γc gene, EDγ cells, and ED40515(–) cells transduced with the indicated vectors were stimulated with IL-2 for 30 min, and STAT5 phosphorylation in each cell line was detected by a STAT5 phosphospecific mAb.

FV vector plasmids were constructed as previously described [13], [18], [19]. In brief, a 631-bp *BspE*I and *Tth111*I restriction fragment from the ubiquitously acting chromatin-opening element promoter from the human HNRPA2B1-CBX3 locus (UCOE631) was isolated and inserted into the pΔΦ vector plasmid together with either the human γc (*IL2RG*) or enhanced green fluorescent protein (EGFP) complementary DNA (cDNA) to generate FV-IL2RG and FV-EGFP (Fig. 1A).

FV particles were produced by transfecting 293T cells with the resultant gene transfer vector plasmids and three helper plasmids (pCiGS, pCiPS, and pCiES) using FuGENE HD (Roche Applied Science), as previously described [13]. Culture supernatants were harvested after 48 hours and concentrated by ultracentrifugation.

A γc RV vector was constructed by inserting the human γc cDNA into the multi-cloning site of pMX-IRES-EGFP. Retroviral particles were produced by transfecting into amphotropic retrovirus packaging cells (PLAT-A cells) with pMx-IL2RG-IRES-EGFP using FuGENE HD. Culture supernatants containing the RV vector particles were harvested after 48 hours.

### Cell lines

A human T cell line, ED40515(–) [20], which lacks γc expression, and an ED40515(–)-derived transfectant with a γc gene, EDγ, were described previously [21], [22]. These cell lines were cultured in RPMI1640 medium supplemented with 10% FCS.

### Mice

The γc-KO mice were previously reported [23]. γc-KO mice on a NOD/scid background [24] were obtained from the Central Institute for Experimental Animals (CIEA, Kawasaki, Japan). They were housed under specific pathogen-free conditions in individually ventilated cages and supplied with sterile food, water, and bedding. All procedures were performed according to protocols approved by the Institutional Committee for the Use and Care of Laboratory Animals of Tohoku University (2011MA139).

### Vector transduction and BMT

Lineage marker depleted (Lin^−^) cells were purified from the bone marrow cells of male γc-KO mice using magnetic cell sorting. The purified cells were exposed to FV vectors in StemSpan medium (StemCell Technologies Japan, Tokyo) supplemented with stem cell factor (50 ng/ml), IL-3 (5 ng/ml), Flt-3 ligand (5 ng/ml), and IL-6 (10 ng/ml) (all from Wako Pure Chemical Industries, Tokyo, Japan) on CH-296 (Retronectin; Takara Shuzo, Otsu, Japan)-coated plates for 16 hours. The transduced cells (1–3×10^6^) were then transplanted intravenously into 120 rad-irradiated female γc-KO mice on a NOD/scid background of 6–8 weeks of age. Eight to 12 weeks after BMT, and the peripheral blood cells and splenocytes were analyzed.

The ED40515 cell lines were similarly transduced with FV or RV vectors, except that cytokines were not added.

### Immunofluorescence staining

For fluorescence-activated cell sorting (FACS) analysis, peripheral blood cells and splenocytes were collected from the mice. After removing erythrocytes with a lysing buffer, the cells were stained with anti-CD3-allophycocyanin (APC), anti-CD4-APC, anti-CD8-phycoerythrin (PE), anti-NK1.1-PE, anti-B220-APC, and/or anti-IgM-PE monoclonal antibodies (mAbs). Stained cells were analyzed with a FACS CantoII analyzer using the FACS Diva software (BD Biosciences, San Jose, CA).

### Phosphorylated STAT5 detection

The IL-2-induced phosphorylation of STAT5 was detected by Western blotting as previously described [22]. In brief, cells were stimulated with 100 ng/ml IL-2 for 30 min at 37°C, collected, and lysed with a lysis buffer (20 mM Tris-HCl (pH 7.4), 150 mM NaCl, 2 mM EDTA, 1% NP-40, 50 mM NaF, 1 mM Na_3_VO_4_, and Protease Inhibitor Cocktail (Sigma-Aldrich Japan, Tokyo)). The protein lysates were separated by electrophoresis, transferred onto a polyvinylidene fluoride membrane, and blotted with STAT5 phosphospecific (pY694) antibodies (Cell Signaling Technology, Japan). Bound primary Abs were detected by a horseradish peroxidase-conjugated anti-rabbit IgG Ab followed by an enhanced chemiluminescence (ECL) detection reagent.

### T cell proliferation and cytokine production

Spleen cells (1×10^5^ cells/well) were stimulated with plate-coated anti-CD3 mAb (clone 2C11; 0.5 or 10 µg/ml) and/or 100 ng/ml recombinant human IL-2. T cell proliferation was measured after 48 hours by [^3^H] thymidine incorporation and scintillation counting in triplicate. The stimulation index was calculated as the ratio of the incorporated radioactivity (cpm) of splenocytes from mice treated with FV-IL2RG-treated HSCs to that of splenocytes from mice treated with FV-EGFP-treated HSCs. IL-2 and IFN-γ production was assessed with an OptiEIA ELISA kit (BD Biosciences) after 24 hours and 72 hours, respectively, of stimulation, following the manufacturer's specifications.

### Analysis of vector-insertion sites by ligation-mediated PCR

To determine the vector-integration sites, linker-mediated PCR was performed on genomic DNA isolated from γc-transduced ED40515(−) cells as previously described [13], [25]. Briefly, the genomic DNA was digested with *Mse*I and *Pst*I, and the fragments were ligated to an MseI linker (5′-GTAATACGACTCACTATAGGGCTCCGCTTAAGGGACGAGGCGAATTCCCTGAT-3′, 5′-PO4-TAGTCCCTTAAGCGGAG-NH2-3′). PCR was then performed with a linker-specific primer 5′-GTAATACGACTCACTATAGGGC-3′, and an FV long-terminal repeat-specific primer 5′-GTCTATGAGGAGCAGG AGTA-3′ or an RV long-terminal repeat-specific primer 5′-TAACCAATCAGTTCGCTTCTCGCTT-3′. A nested PCR was then performed using a linker-specific primer 5′-AGGGCTCCGCTTAAGGGAC-3′, and an FV long-terminal repeat-specific primer 5′-CCTCCTTCCCTGTAATACTC-3′ or an RV long-terminal repeat-specific primer 5′-CTCAATAAAAGAGCCCACAACCCC-3′. The PCR products were subcloned into pCR2.1 using the TOPO cloning kit (Life Technologies Japan, Tokyo), and the vector/DNA junction sites were sequenced with an ABI 3100 Genetic Analyzer (Applied Biosystems). The recovered vector/DNA junctions were matched to the human genome using the BLAT software program and each insertion locus was identified. Since integration sites of conventional RV vectors are preferentially found within 15 kb of transcriptional start sites (TSS) [26], [27], we calculated the percentages of all integration sites within 15 kb of TSS. The integration frequency was calculated as previously described [28]. In brief, FV vector integration sites were mapped relative to RefSeq gene transcription start sites, binned into different size sequence windows, and plotted as the percent of all integrations per kb. Genes within 30 kb of the integration sites were also compared to the list of annotated cancer genes in the Atlas of Genetics and Cytogenetics in the Oncology and Hematology database (http://atlasgeneticsoncology.org/).

### Statistical analysis

Statistical analysis was performed using χ^2^-test or Student's t-test. P-values <0.05 were considered significant.

## Results

### FV vector performance in human T cells

We constructed two FV vectors, FV-IL2RG and FV-EGFP, to express human γc and EGFP, respectively, both of which were driven by the ubiquitously acting chromatin-opening element promoter (Fig. 1A, and see Materials and Methods). This promoter consists of a methylation-free CpG island without classic enhancer activity. To evaluate the function of the FV-IL2RG vector-expressed γc chain, we infected ED40515(−), a human T cell line lacking γc [21], [22], with the FV vectors. A flow cytometric analysis detected clear γc expression on the surface of the FV-IL2RG-treated cells (Fig. 1B). Western blot analysis showed the phosphorylation of Stat5 upon IL-2 stimulation, reflecting the activation of intracellular signaling through the vector-mediated γc (Fig. 1C), and indicating the expression of functional γc. These results indicated that FV vectors can effectively transfer and express the γc gene in human T cells.

### Profile of provirus integration sites in human T cells

**Figure 2:**
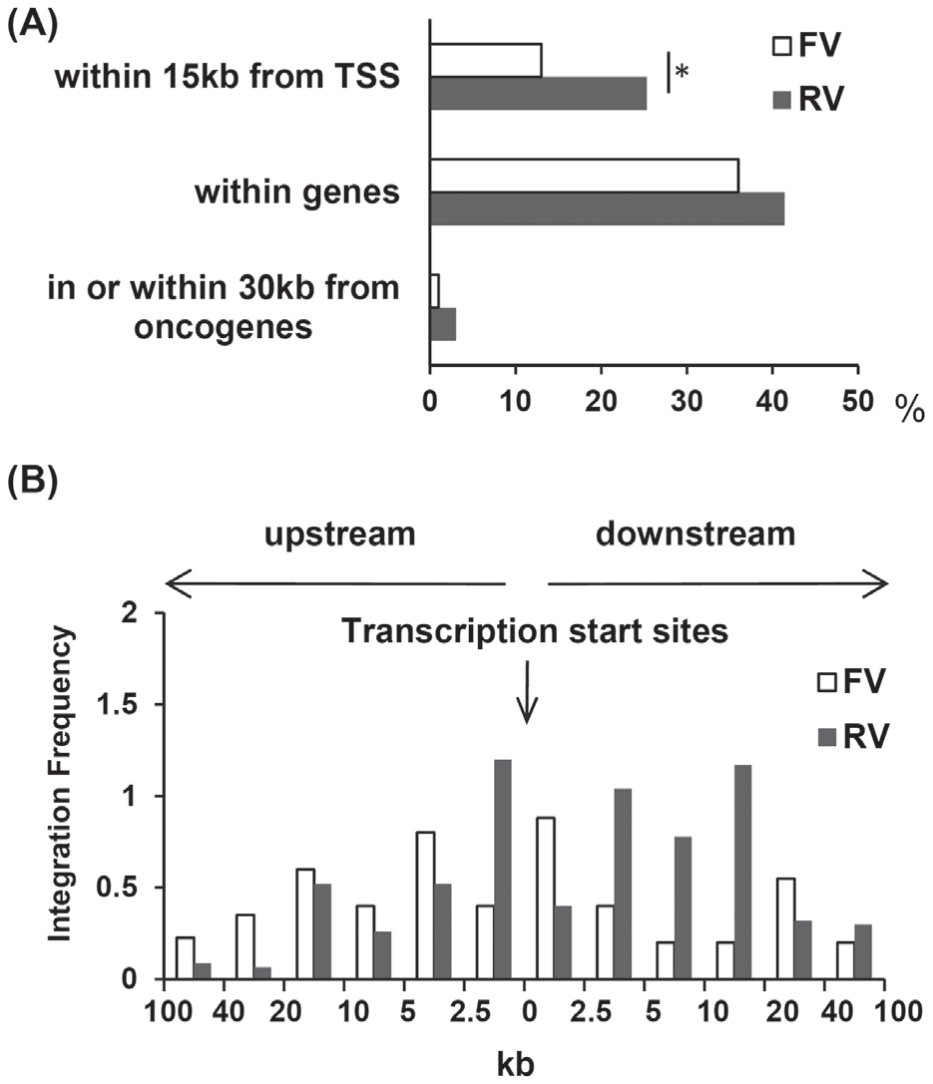
Profile of provirus integration in transduced cells. (**A**) Position of FV and RV integration sites. The percentage of all integration sites within 15 kb of transcriptional start sites, within genes that contain putative microRNA genes, and within 30 kb of oncogenes is shown for FV vector- or RV vector-treated cells. *p<0.05, χ^2^-test. (**B**) A 100-kb window centered on TSS in the RefSeq database is shown. Relative frequencies of FV and RV vector integrations in each interval were calculated by dividing the percentage of integration b the indicated interval length.

We identified 100 independent integration sites of the FV or RV provirus in ED40515(−) cells infected with each vector, using a standard linker-mediated PCR analysis. Fig. 2A shows that the frequency of integration sites located within gene transcriptional units was comparable between the FV (36%) and RV (42%) vectors. However, the FV integration sites showed a significantly lower likelihood (13%) of being located immediately up or downstream of transcriptional start sites than the RV integration sites (25%) (Fig. 2A). Furthermore, the FV integration sites in the human T cells showed only a modest preference for regions near transcriptional start sites compared to RV integration sites (Fig. 2B), consistent with the results of previous studies [13], [25], [29].

In addition, we examined the cancer-related genes located within 30 kb of the FV and RV integration sites because all the RV insertion sites in gene therapy-related leukemia were shown within gene or within 30 kb of TSS of LMO-2 or BMI1 [30]. One of the 100 FV integration sites was detected inside the cancer-related gene, TCF12, which is known as a negative regulator of cell proliferation, whereas three of the RV integration sites were detected within cancer-related genes, (Klf5, NUMB, and FHIT) all of which are known leukemia-related genes.

Collectively, these results suggest that FV vectors might have a lower risk of vector-mediated genotoxicity than conventional RV vectors.

### In vivo assessment of T cell restoration after FV-mediated gene therapy

To evaluate the efficacy of FV vector-mediated γc expression *in vivo*, we performed BMT experiments. Bone marrow Lin^−^ cells from γc-KO mice were transduced with FV-IL2RG or FV-EGFP vectors. The cellular transduction efficiency of both vectors was between 33 and 40%. The FV vector-infected cells were intravenously transplanted into γc-KO mice on a NOD/scid background. Since older γc-KO mice on a C57BL/6 background were found to contain CD3^+^CD4^+^ cells (data not shown), γc-KO mice on a NOD/scid background were deemed a more suitable recipient for evaluating T cell reconstitution.

**Figure 3:**
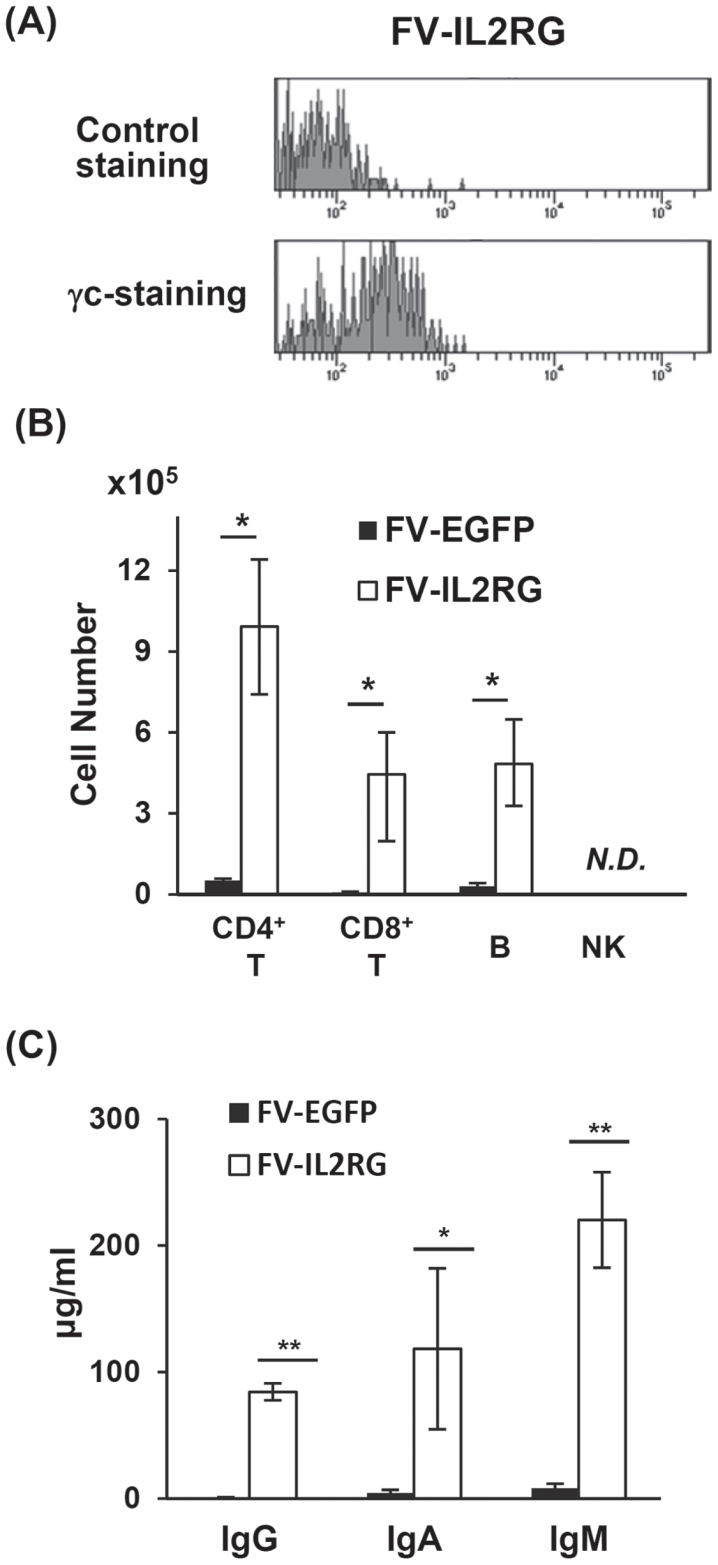
Reconstitution of T and B cells. (**A**) Cell-surface expression of γc on peripheral CD8^+^ T cells in γc-KO mice treated with FV-IL2RG-treated HSCs. The upper and lower panels show isotype-control and γc-specific stainings, respectively. (**B**) The absolute numbers of CD4^+^ T, CD8^+^ T, sIgM^+^ B, and NK cells in the spleen of γc-KO mice treated with FV-EGFP and FV-IL2RG (n = 4 in each group). *N.D.*, not detectable. (**C**) Serum IgM, IgG, and IgA in FV-IL2RG-treated mice. Serum levels of IgM, IgG, and IgA were measured by ELISA. Results shown are the mean ± SD of the stimulation index from 4 mice in each group. *p<0.05 and **p<0.01, Student t-test.

Eight weeks after transplantation, T cells emerged in the peripheral blood of the FV-IL2RG-treated group, and the clear expression of γc was confirmed on CD8^+^ T cells (Fig. 3A). Gene therapy mice showed the recovery of CD4 and CD8 T cells as well as B220^+^ IgM^+^ B cells in spleen (in Figure 3B) although we could not detect NK cells. In addition, serum levels of IgM, IgG, and IgA were significantly elevated in FV-IL2RG-treated mice (Fig. 3C). These results indicate that HSCs transduced with FV-IL2RG have the potential for normal B and T cell differentiation.

**Figure 4:**
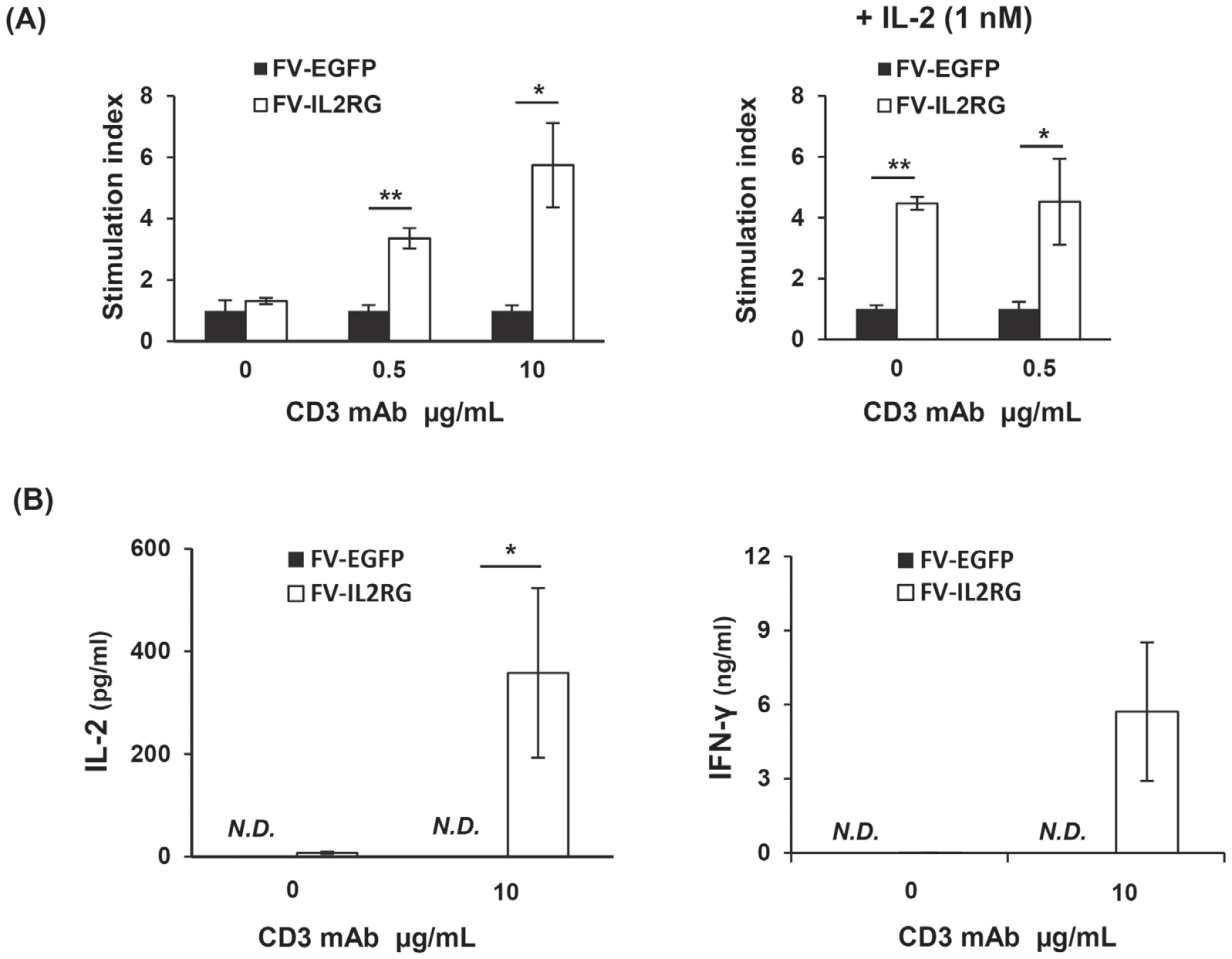
In vitro function of reconstituted T cells. (**A**) Splenocytes from recipient mice were stimulated with an anti-CD3 mAb at the indicated dose in the presence (right) or absence (left) of IL-2. Results shown are the mean ± SD of the stimulation index from 4 or 5 mice in each group. *p<0.05 and **p<0.01, Student t-test. (**B**) IL-2 and IFN-γ production by reconstituted T cells. Splenocytes from recipient mice were stimulated with an anti-CD3 mAb at the indicated dose. Culture supernatants were collected 24 and 72 hours after stimulation. Concentration of each cytokine in the supernatant was measured by ELISA. Results shown are the mean ± SD from 4 mice in each group. *p<0.05, Student t-test. *N.D.*, not detectable.

We next investigated the functional capacity of the reconstituted T cells. Splenocytes from FV-IL2RG-treated mice showed proliferative responses following treatment with an anti-CD3 mAb, while those from the FV-EGFP-treated group did not (Fig. 4A). IL-2 stimulation induced proliferation through γc-transduced signals in T cells from FV-IL2RG-treated mice although stimulation with anti-CD3 mAb plus IL-2 did not enhance T cell proliferation compared to anti-CD3 mAb alone (Fig. 4A). The reconstituted T cells also produced IL-2 and IFN-γ upon stimulation with anti-CD3 mAb (Fig. 4B), indicating the functional restoration of T cells. Collectively, FV vector-mediated γc gene transfer was demonstrated to restore T and B cell differentiation and function *in vivo*.

## Discussion

Vector-mediated insertional mutagenesis is a critical problem associated with conventional RV-mediated gene therapy treatments for SCID-X1. To address this issue, we developed a modified FV vector carrying the human γc gene, and evaluated the vector-mediated insertional mutagenesis as well as the *in vivo* T, B, and NK cell reconstitution. Our findings demonstrated that the integration sites of the FV vector were significantly less likely to be located within or near transcriptional start sites compared to those of a conventional RV vector, suggesting that the FV vector had a lower risk for insertion-mediated genotoxicity. We also showed the successful reconstitution of functionally active T and B cells after the transplantation of HSCs containing a γc-FV vector into γc-KO recipient mice. This is the first reported use of an FV vector in a gene therapy mouse model of SCID-X1.

Previous studies showed that the use of classical RV vector for SCID-X1 gene therapy resulted in the development of leukemia in a number of patients [4], [5]. The leukemogenesis associated with these vectors is likely to be due to inappropriate vector insertion in or near proto-oncogenes. Consistent with previous reports that FV vectors have a more random genomic integration pattern than RV vectors [28], our data indicated that the FV vector integration sites were less likely to be near transcription start sites than those of RV vectors (Fig. 2A, 2B). In addition, three integration sites of the RV vector were found within cancer-related genes (Klf5, NUMB, and FHIT), all of which are associated with leukemogenesis or leukemia progression [31]–[33]. Although one FV vector integration site was found within a cancer-related gene, TCF12, no evidence showing a relationship between TCF12 and leukemogenesis has been reported. These results are consistent with the accepted theory that FV is non-pathogenic for humans [8], [9], and support the notion that FV vectors may be safer than conventional RV vectors.

The main objective of SCID-X1 gene therapy is the robust reconstitution of T, B, and NK cells. However, in the present study, use of the FV-IL2RG vector failed to reconstitute the NK cell population when monitored up to 4 months after gene therapy. One possible explanation for this deficiency may be that the human γc gene is not entirely compatible with the mouse system. Consistent with our findings, impaired NK cell reconstitution in mouse SCID-X1 gene therapy using the human γc gene was previously reported [34]; however, gene therapies in mouse models with RV vectors carrying the mouse γc gene were shown to reconstitute NK cells within two months [35]–[37]. NK cell development requires IL-15, while the differentiation and survival of T and B cells require IL-7. Receptors for both cytokines contain the shared γc subunit [1]. Therefore, the human γc might function less effectively as a mouse IL-15 receptor subunit than as a mouse IL-7 receptor subunit. In addition, T cell responses to IL-2 might also be insufficient as additional stimulation of IL-2 did not increase the T cell response with anti-CD3 mAb alone. Namely, the chimeric IL-2 and IL-15 receptors consisting of mouse α and β chains in combination with human γc might not fully function as a physiological mouse IL-2 and IL-15 receptors, respectively. In any case, the use of human γc, rather than the FV vector, is the probable cause of the impaired NK cell reconstitution and possible insufficient T cell function in this study.

We recently reported the successful treatment of a WAS mouse model using gene therapy with an FV vector [13]. Similar to SCID-X1, the gene therapy for WAS requires T cell proliferative and functional restoration. Our studies collectively support the potential for modified FV vector-mediated gene therapy for the successful treatment of T cell immunodeficiencies. Moreover, FV vectors have also been shown to be effective for the treatment of red blood cell and granulocyte disorders in mouse models [14]–[17]. Based on their broad host range and ability to efficiently transduce HSCs, FV vectors may be applicable in gene therapies for many different hematopoietic disorders.

Over the last decade, novel modifications of RV vectors have been developed to overcome the problems associated with SCID-X1 gene therapy. However, it is still controversial which vector is the most favorable for safety. FV vectors have the potential to increase the efficacy of HSC-based gene therapies and to reduce the risk of genotoxicity. Although further studies are necessary, our data support the potential clinical application of FV vectors in gene therapy for SCID-X1 in the future.
